# Supplementary material for: Genome-Wide Identification, Characterization and Expression Analysis of Plant Nuclear Factor (NF-Y) Gene Family Transcription Factors in Saccharum spp
Source: Genes (Basel). 2023 May 25;14(6):1147. doi: 10.3390/genes14061147 (PMC10298139; doi:10.3390/genes14061147)
Supplement: Supplementary file 1 [file genes-14-01147-s001.zip › Supplementary Figures.pdf]

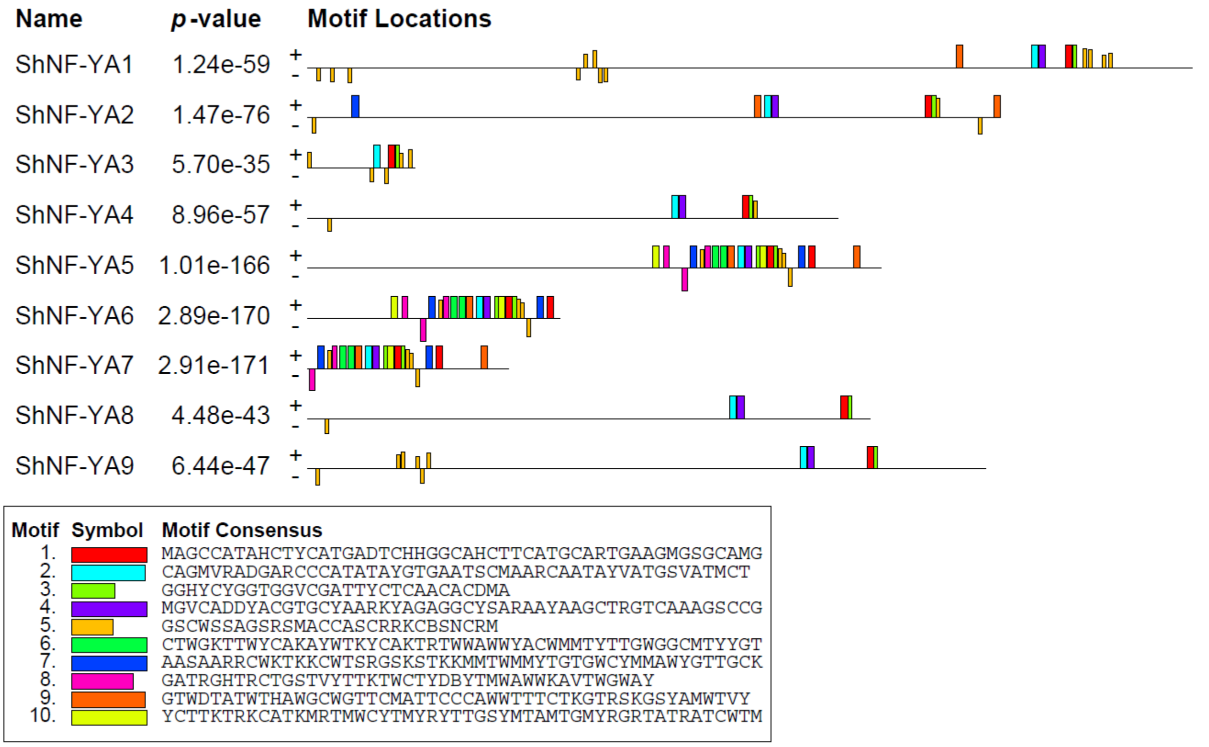
Name
p-value
Motif Locations
ShNF-YA1 1.24e-59
ShNF-YA2 1.47e-76
ShNF-YA3 5.70e-35
ShNF-YA4 8.96e-57
ShNF-YA5 1.01e-166
ShNF-YA6 2.89e-170
ShNF-YA7 2.91e-171
ShNF-YA8 4.48e-43
ShNF-YA9 6.44e-47
Motif Symbol Motif Consensus
1. MAGCCATAHCTYCATGADTCHHGGCAHCTTCATGCARTGAAGMGSGCAMG
2. CAGMVRADGARCCCATATAYGTGAATSCMAARCAATAYVATGSVATMCT
3. GGHYCYGGTGGVCGATTYCTCAACACDMA
4. MGVCADDYACGTGCYAARKYAGAGGCYSARAAYAAGCTRGTCAAAGSCCG
5. GSCWSSAGSRSMACCASCRRKCBSNCRM
6. CTWGKTTWYCAKAYWTKYCAKTRTWWAWWYACWMMTYTTGWGGCMTYYGT
7. AASAARRCWKTKKCWTSRGSKSTKKMMTWMMYTGTGWCYMMAWYGTTGCK
8. GATRGHTRCTGSTVYTTKTWCTYDBYTMWAWWKAVTWGWAY
9. GTWDTATWTHAWGCWGTTCMATTCCCAWWTTTCTKGTRSKGSYAMWTVY
10. YCTTKTRKCATKMRTMWCYTMYRYTTGSYMTAMTGMYRGRTATRATCWTM

(a)

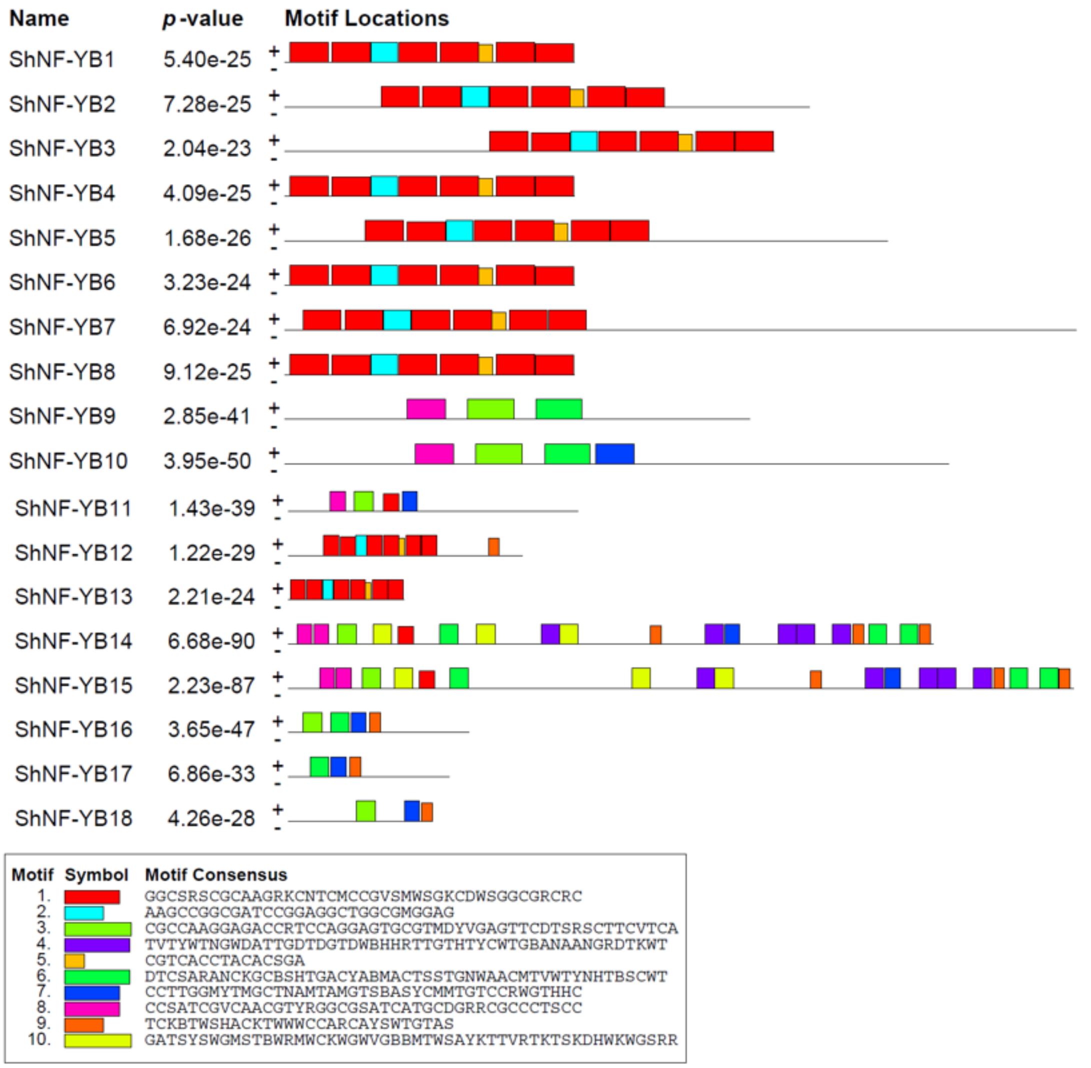
Name
p-value
Motif Locations
ShNF-YB1 5.40e-25
ShNF-YB2 7.28e-25
ShNF-YB3 2.04e-23
ShNF-YB4 4.09e-25
ShNF-YB5 1.68e-26
ShNF-YB6 3.23e-24
ShNF-YB7 6.92e-24
ShNF-YB8 9.12e-25
ShNF-YB9 2.85e-41
ShNF-YB10 3.95e-50
ShNF-YB11 1.43e-39
ShNF-YB12 1.22e-29
ShNF-YB13 2.21e-24
ShNF-YB14 6.68e-90
ShNF-YB15 2.23e-87
ShNF-YB16 3.65e-47
ShNF-YB17 6.86e-33
ShNF-YB18 4.26e-28
Motif Symbol Motif Consensus
1. GGCSRSCGCAAGRKCNTCMCCGVSMWSGKCDWSGGCGRCRC
2. AAGCCGGCGATCCGGAGGCTGGCGMGGAG
3. CGCCAAGGAGACCRTCCAGGAGTGCGTMDYVGAGTTCDTSRSCTTCVTCA
4. TVTYWTNGWDATTGDTDGTDWBHHRTTGTHTYCWTGBANAANGRDTKWT
5. CGTCACCTACACSGA
6. DTCSARANCKGCBSHTGACYABMACTSSTGNWAACMTVWTYNHTBSCWT
7. CCTTGGMYTMGCTNAMTAMGTSBASYCMMTGTCCRWGTHHC
8. CCSATCGVCAACGTYRGGCGSATCATGCDGRRCGCCCTSCC
9. TCKBTWSHACKTWWWCCARCAYSWTGTAS
10. GATSYSWGMSTBWRMWCKWGWVGBBMTWSAYKTTVRTKTSKDHWKWGSRR

(b)

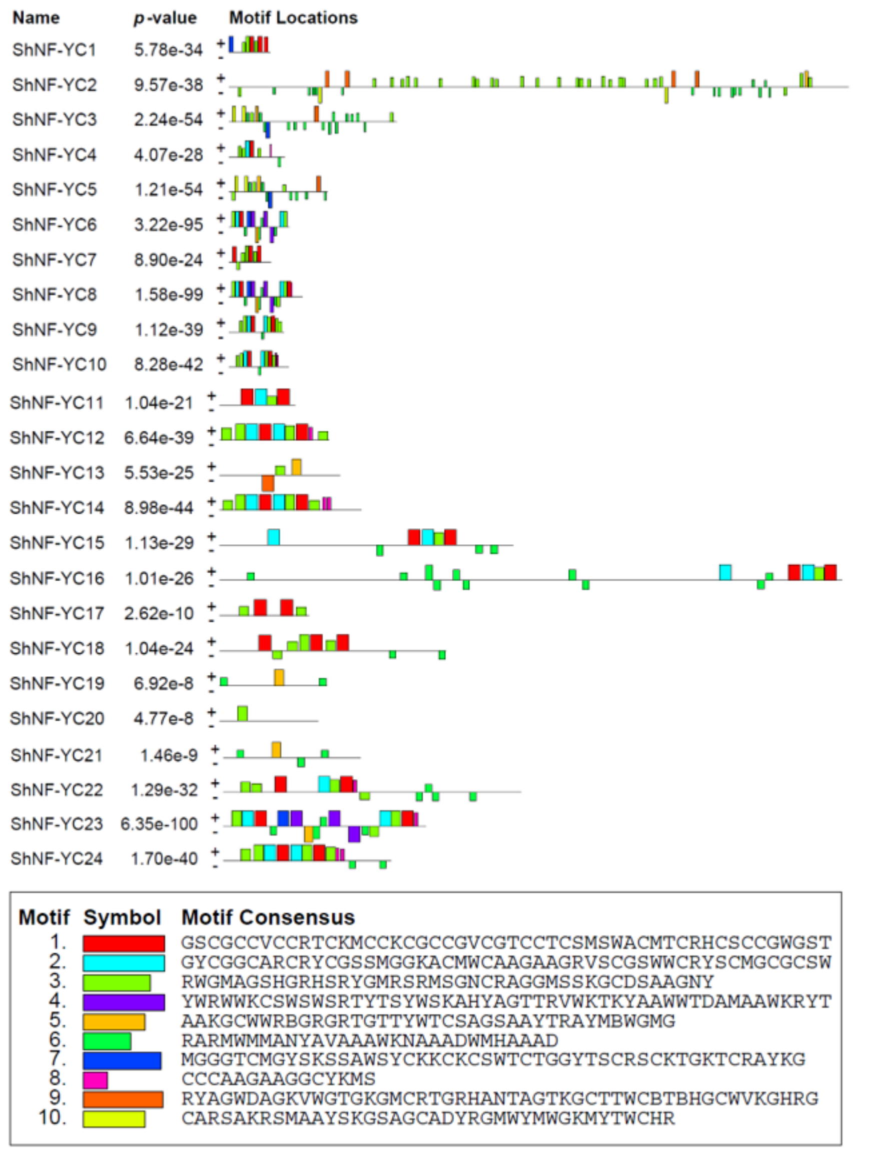

(c)

Figure S1. Motifs predicted in ShNF-Y genes; (a) ShNF-YA; (b) ShNF-YB; (c) ShNF-YC

ShNF-YA

ShNF-YA1

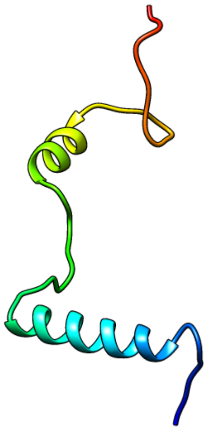

ShNF-YA2

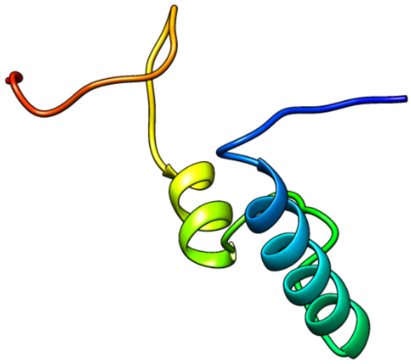

ShNF-YA3

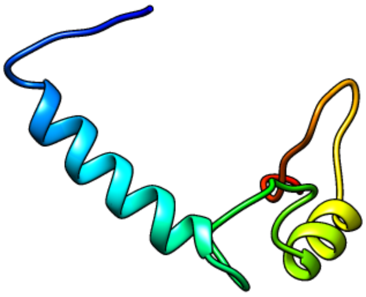

ShNF-YA4

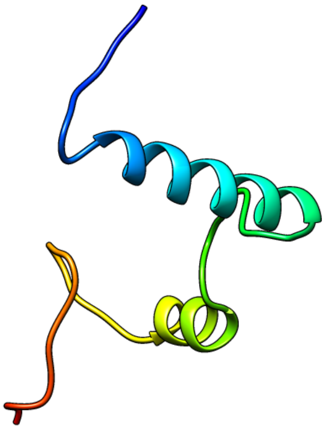

ShNF-YA5

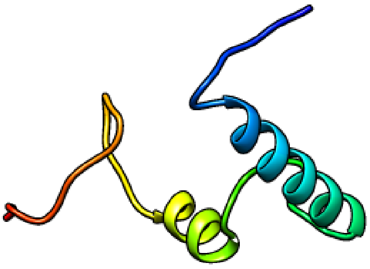

ShNF-YA6

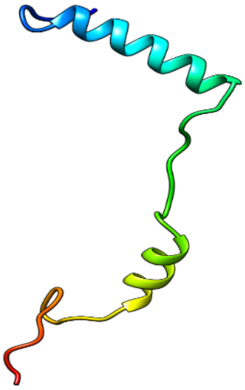

ShNF-YA7

ShNF-YA8

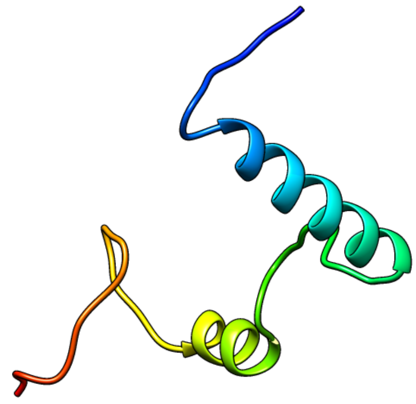

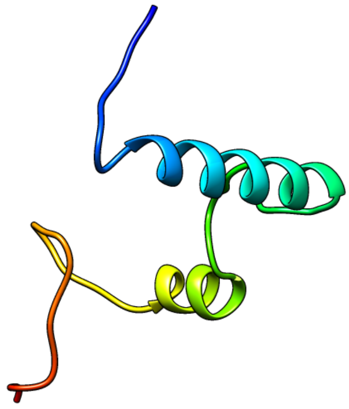

ShNF-YA9

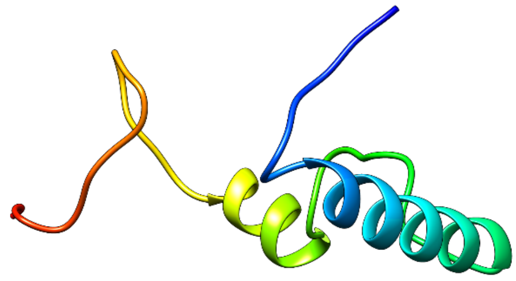

(a)

ShNF-YB proteins

ShNF-YB1

ShNF-YB2

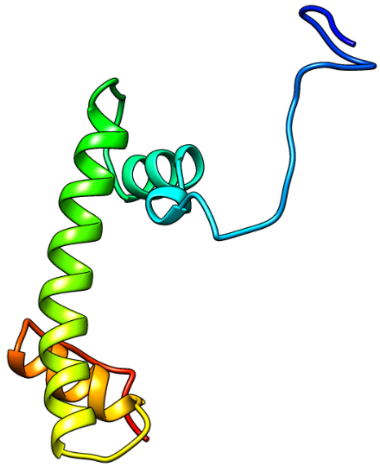

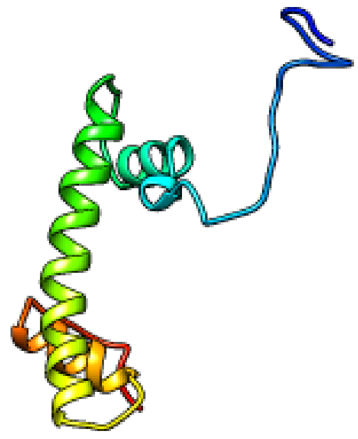

ShNF-YB3

ShNF-YB4

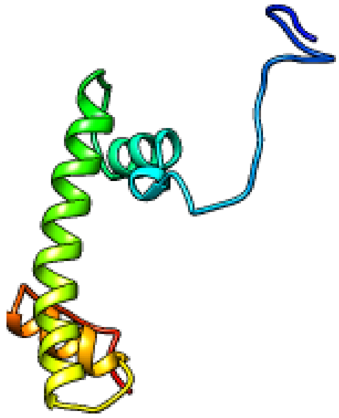

ShNF-YB5

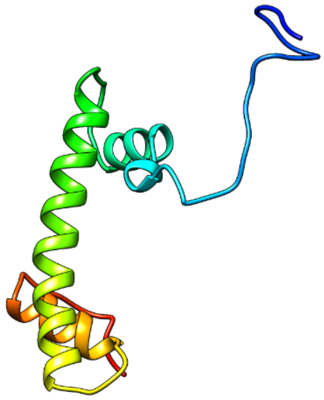

ShNF-YB6

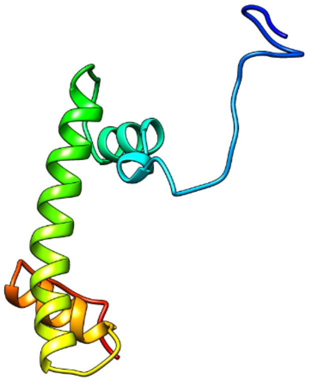

ShNF-YB7

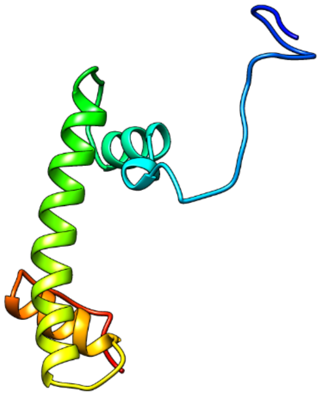

ShNF-YB8

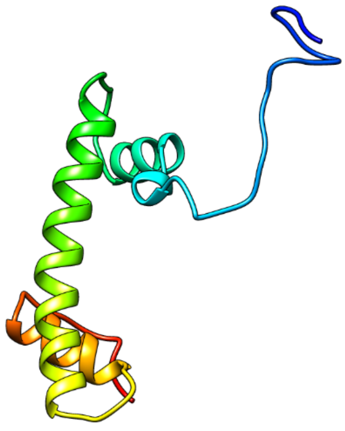

ShNF-YB9

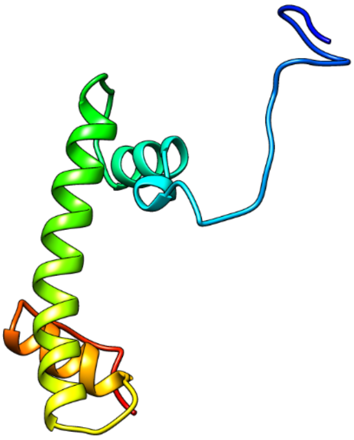

ShNF-YB10

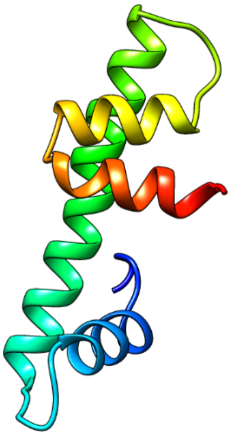

ShNF-YB11

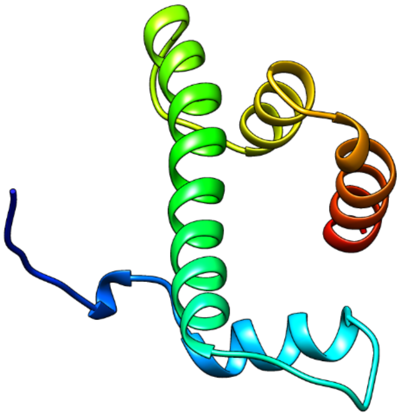

ShNF-YB12

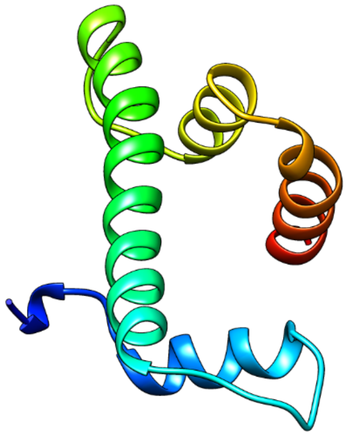

ShNF-YB13

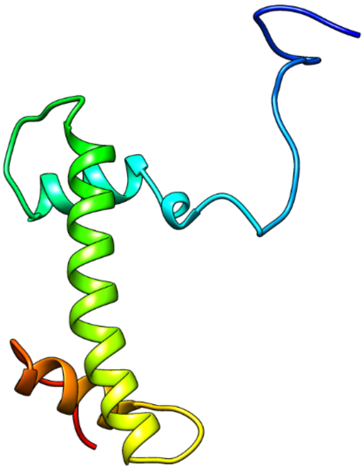

ShNF-YB14

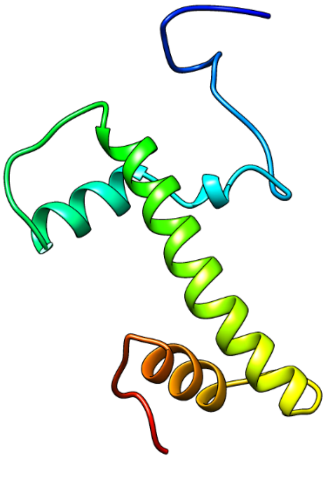

ShNF-YB15

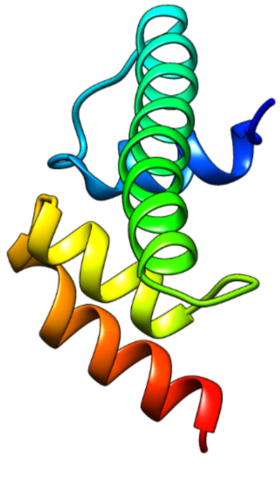

ShNF-YB16

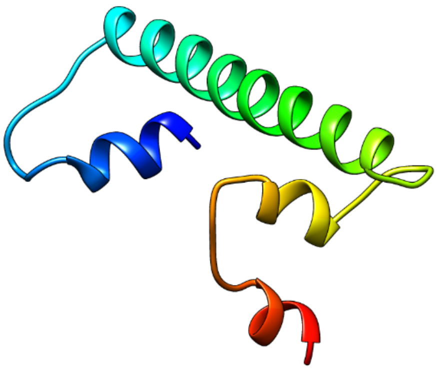

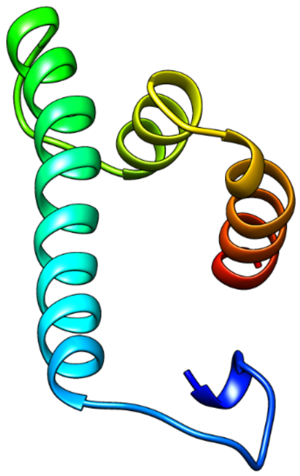

ShNF-YB17

ShNF-YB18

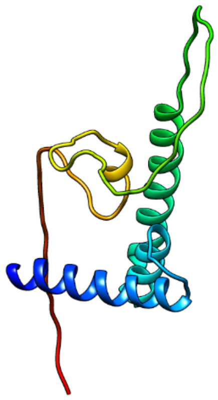

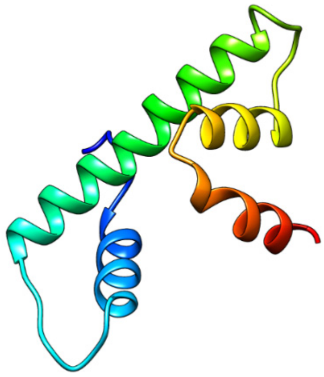

(b)

ShNF-YC proteins

ShNF-YC1

ShNF-YC2

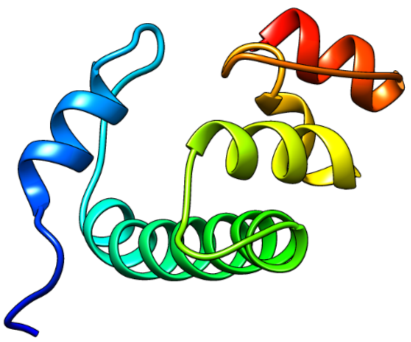

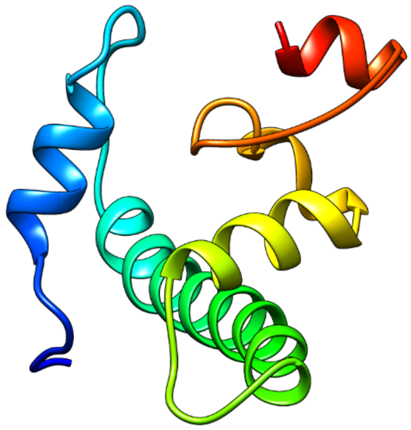

ShNF-YC3

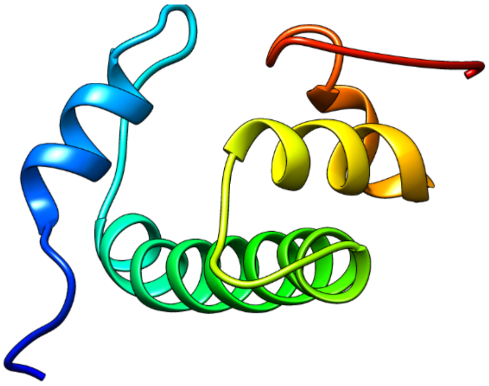

ShNF-YC4

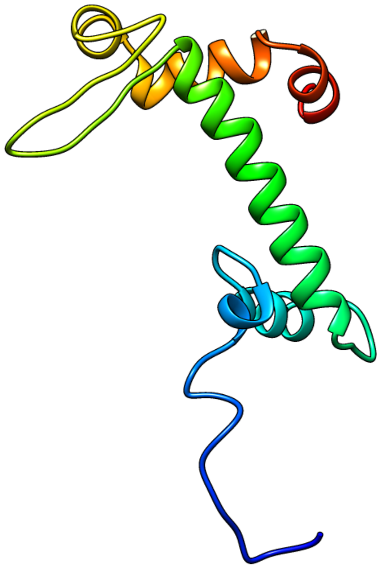

ShNF-YC5

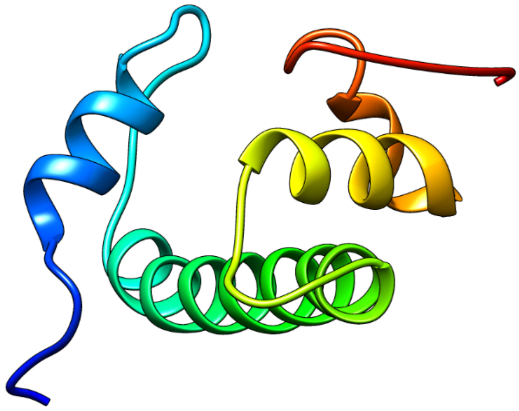

ShNF-YC6

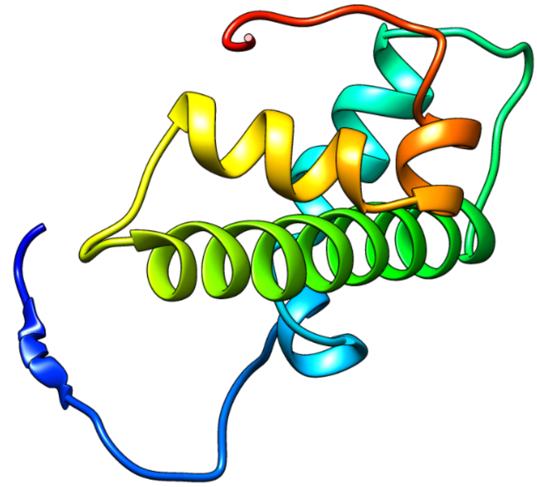

ShNF-YC7

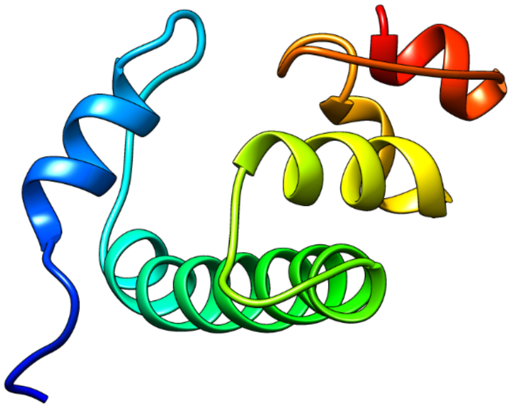

ShNF-YC8

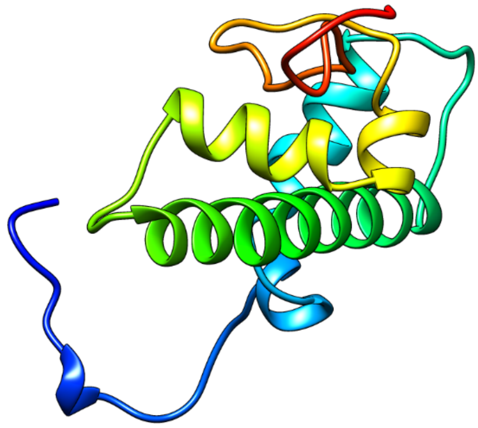

ShNF-YC9

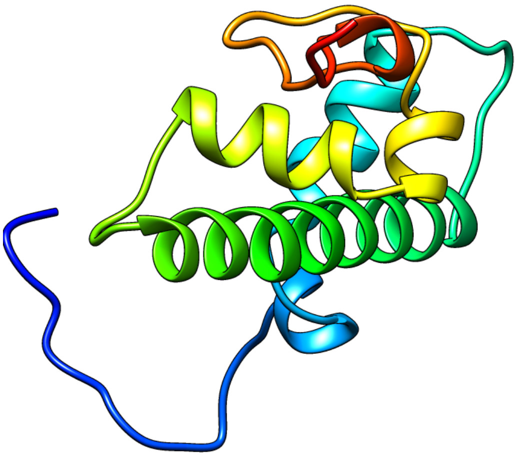

ShNF-YC10

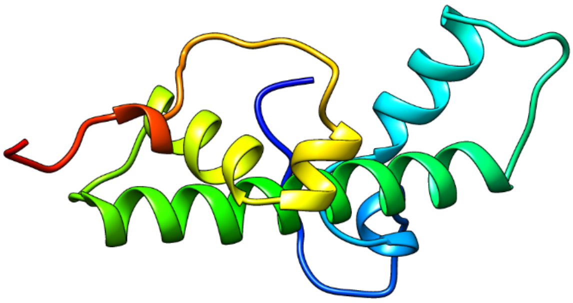

ShNF-YC11

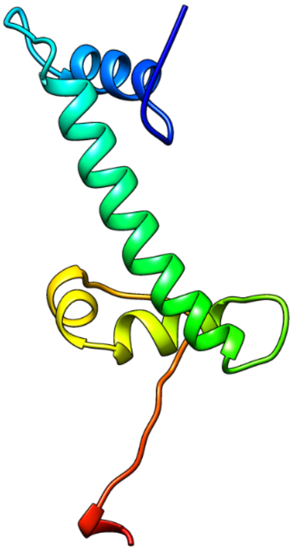

ShNF-YC12

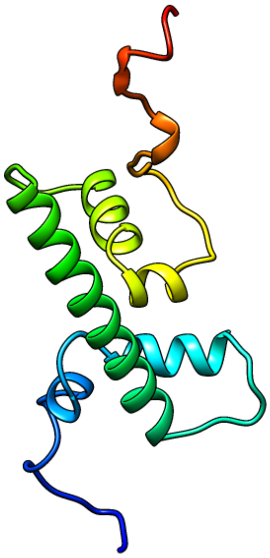

ShNF-YC13

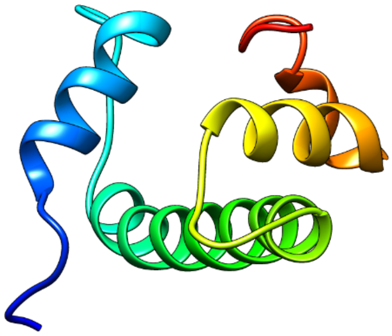

ShNF-YC14

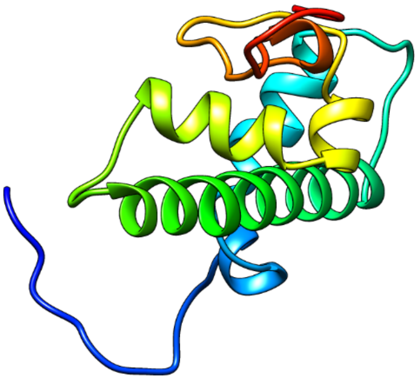

ShNF-YC15

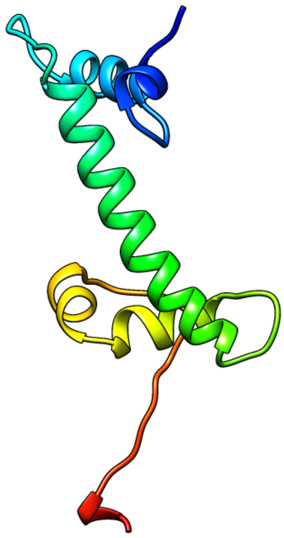

ShNF-YC16

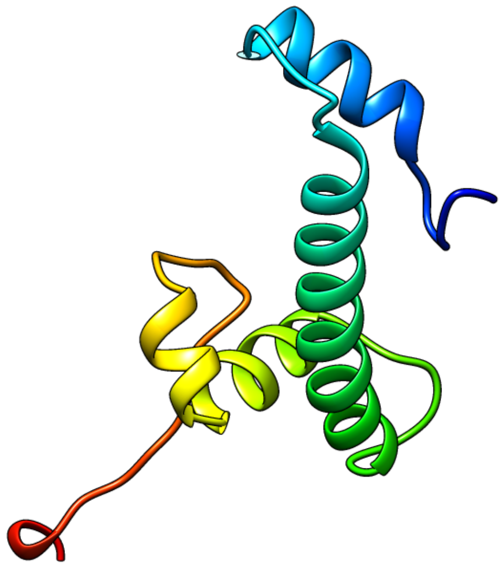

ShNF-YC17

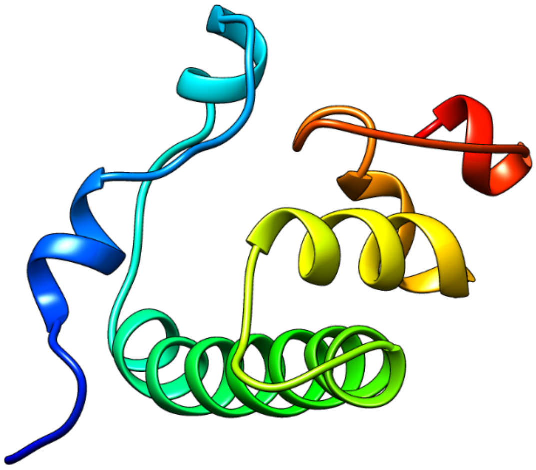

ShNF-YC18

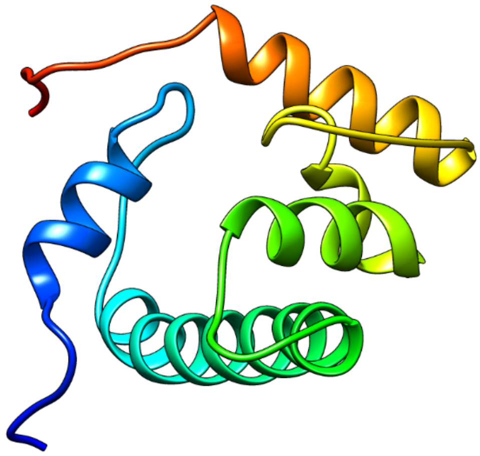

ShNF-YC19

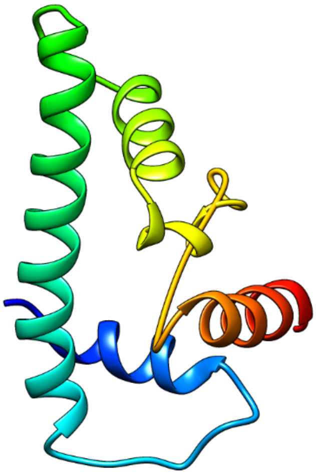

ShNF-YC20

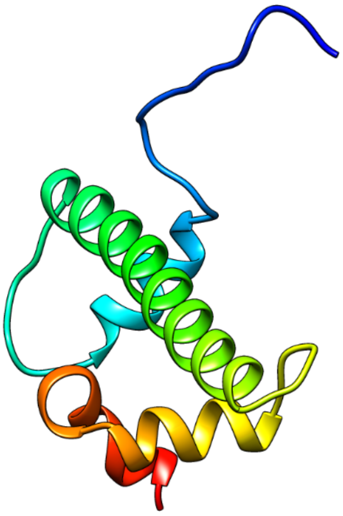

ShNF-YC21

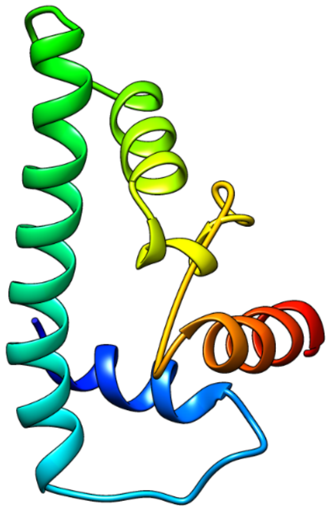

ShNF-YC22

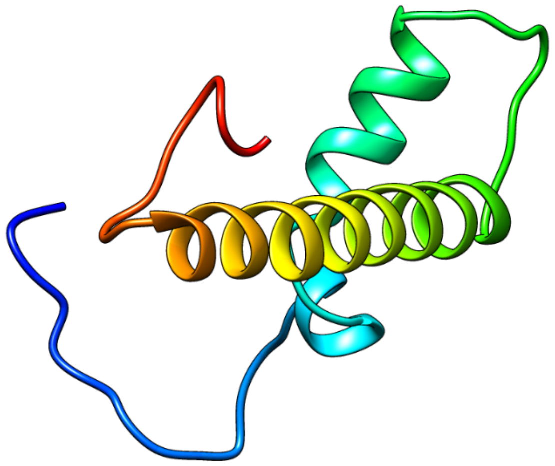

ShNF-YC23

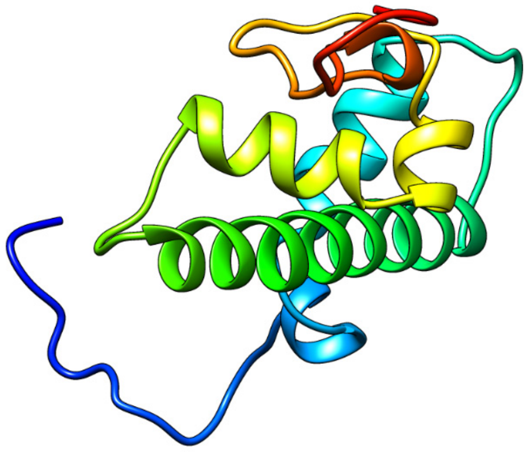

ShNF-YC24

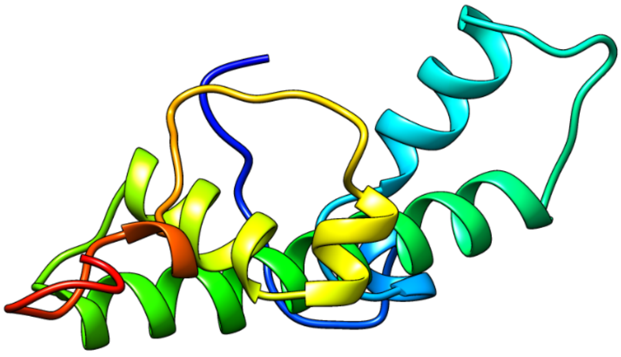

(c)

Figure S2. Three dimensional structures models of ShNF-Y proteins; (a) ShNF-YA; (b) ShNF-YB; and (c) ShNF-YC.
