# Supplementary material for: Genome-Wide Identification, Characterization and Expression Analysis of Plant Nuclear Factor (NF-Y) Gene Family Transcription Factors in Saccharum spp
Source: Genes (Basel). 2023 May 25;14(6):1147. doi: 10.3390/genes14061147 (PMC10298139; doi:10.3390/genes14061147)
Supplement: Supplementary file 1 [file genes-14-01147-s001.zip › Supplementary Tables S1 and S2.pdf]

Table S1. Primers used in real time quantitative expression analysis of *ShNF-Y* genes.

**Genes**	Forward Primer	Reverse Primer
ShNF-YA1	CCTGTCGCCAGAGGAGA	GACTCCAAATTCCCTTGGAAGA
ShNF-YA3	CGTTTCGAGCTTGGCATTG	GGCTCCGTAGAAGGGATAGTA
ShNF-YA5	CCAGACCAGAGGAAGGGA	CGGGAGAGGAAGACGAAGA
ShNF-YA9	GCTCATCAAGGTAAAGGGAAGA	CCATAGTGTTGGTCAGCATAAGA
ShNF-YB2	TCTCACCTGGGCTGCTAAA	GGGAACAGGGAAGCTTGTTT
ShNF-YB8	AGCAATGTCCAGCGAAGAA	GGATTACCTGCCGCATGA
ShNF-YB9	TCTACCTCCACCGCTACC	GGACTTGAGTGGGTGGTG
ShNF-YB16	ATGAGTACGTCGTGCAATTCT	TCGCAAAGCCAGCGATTA
ShNF-YB18	CCATTGCCTGTAGCACATCTA	AGGACTTGGCGCATGATAAC
ShNF-YC7	ATTCCCACCACTCAAGTTCTC	GCACCCTTCTTCGCCTT
ShNF-YC9	TGTTTGCGCTTTGCTTCG	ATGCGGGTCTTCTTGTTGT
ShNF-YC14	TACGGTTCATCGCATCCTTATC	GGTGTACAGATCGGGTTGAAA
ShNF-YC18	GCGCGCATCAAGAAGATCA	GAGCTCGGCGACGAAGA
ShNF-YC24	CTGCGATGAGCTAAGGAAGTT	AGCCAATCTTGGCCTTATCC

Table S2. Subcellular localization and gene ontology of ShNF-Y subunits

Gene name	Subcellular localization	Gene Ontology Terms
**NF-YA Subunit**		
ShNF-YA1	nucleus	CCAAT-binding factor complex GO:0016602(IDA)
ShNF-YA2	nucleus	CCAAT-binding factor complex GO:0016602(IDA)
ShNF-YA3	nucleus	CCAAT-binding factor complex GO:0016602(IDA)
ShNF-YA4	nucleus	nucleus GO:0005634
ShNF-YA5	nucleus	nucleus GO:0005634
ShNF-YA6	nucleus	nucleus GO:0005634
ShNF-YA7	nucleus	nucleus GO:0005634
ShNF-YA8	nucleus	nucleus GO:0005634
ShNF-YA9	nucleus	CCAAT-binding factor complex GO:0016602(IDA)
**NF-YB Subunit**		
ShNF-YB1	nucleus	nucleolus GO:0005730(IEA); nucleosome GO:0000786(IEA); thylakoid GO:0009579(IEA); vacuole GO:0005773(IEA)
ShNF-YB2	nucleus	nucleolus GO:0005730(IEA); nucleosome GO:0000786(IEA); thylakoid GO:0009579(IEA); vacuole GO:0005773(IEA
ShNF-YB3	nucleus	nucleolus GO:0005730(IEA); nucleosome GO:0000786(IEA); thylakoid GO:0009579(IEA); vacuole GO:0005773(IEA)
ShNF-YB4	nucleus	nucleolus GO:0005730(IEA); nucleosome GO:0000786(IEA); thylakoid GO:0009579(IEA); vacuole GO:0005773(IEA)
ShNF-YB5	nucleus	nucleolus GO:0005730(IEA); nucleosome GO:0000786(IEA); thylakoid GO:0009579(IEA); vacuole GO:0005773(IEA)
ShNF-YB6	nucleus	nucleolus GO:0005730(IEA); nucleosome GO:0000786(IEA); thylakoid GO:0009579(IEA); vacuole GO:0005773(IEA)
ShNF-YB7	nucleus	nucleolus GO:0005730(IEA); nucleosome GO:0000786(IEA); thylakoid GO:0009579(IEA); vacuole GO:0005773(IEA)
ShNF-YB8	nucleus	nucleolus GO:0005730(IEA); nucleosome GO:0000786(IEA); thylakoid GO:0009579(IEA); vacuole GO:0005773(IEA)
ShNF-YB9	nucleus	nucleus GO:0005634(IEA)
ShNF-YB10	nucleus	CCAAT-binding factor complex GO:0016602(ISO); chromatin GO:0000785(NAS)
ShNF-YB11	nucleus	CCAAT-binding factor complex GO:0016602(ISO); chromatin GO:0000785(NAS)
ShNF-YB12	nucleus	nucleolus GO:0005730(IEA); nucleosome GO:0000786(IEA); thylakoid GO:0009579(IEA); vacuole GO:0005773(IEA)
ShNF-YB13	nucleus	nucleolus GO:0005730(IEA); nucleosome GO:0000786(IEA); thylakoid GO:0009579(IEA); vacuole GO:0005773(IEA)
ShNF-YB14	nucleus	nucleus GO:0005634(IEA)
ShNF-YB15	nucleus	nucleus GO:0005634(IEA)
ShNF-YB16	nucleus	nucleus GO:0005634(IEA)
ShNF-YB17	nucleus	nucleus GO:0005634(IEA)
ShNF-YB18	nucleus	nucleus GO:0005634(IEA)
**NF-YC Subunit**		
ShNF-YC1	nucleus	nucleosome GO:0000786(IEA); nucleus GO:0005634(IEA)

ShNF-YC2	nucleus	nucleosome GO:0000786(IEA); nucleus GO:0005634(IEA)
ShNF-YC3	nucleus	nucleosome GO:0000786(IEA); nucleus GO:0005634(IEA)
ShNF-YC4	nucleus	nucleosome GO:0000786(IEA); nucleus GO:0005634(IEA)
ShNF-YC5	nucleus	CCAAT-binding factor complex GO:0016602(IDA); nucleoplasm GO:0005654(TAS)
ShNF-YC6	nucleus	CCAAT-binding factor complex GO:0016602(IEA); nucleoplasm GO:0005654(TAS); transcription factor complex GO:0005667(IC)
ShNF-YC7	nucleus	nucleosome GO:0000786(IEA); nucleus GO:0005634(IEA)
ShNF-YC8	nucleus	nucleosome GO:0000786(IEA); nucleus GO:0005634(IEA)
ShNF-YC9	nucleus	nucleosome GO:0000786(IEA); nucleus GO:0005634(IEA)
ShNF-YC10	nucleus	nucleosome GO:0000786(IEA); nucleus GO:0005634(IEA)
ShNF-YC11	nucleus	nucleosome GO:0000786(IEA); nucleus GO:0005634(IEA)
ShNF-YC12	nucleus	CCAAT-binding factor complex GO:0016602(IDA)
ShNF-YC13	nucleus	lipid particle GO:0005811(IDA); nucleosome GO:0000786(IDA); nucleus GO:0005634(IDA); polytene chromosome chromocenter GO:0005701(IDA)
ShNF-YC14	nucleus	lipid particle GO:0005811(IDA); nucleosome GO:0000786(IDA); nucleus GO:0005634(IDA); polytene chromosome chromocenter GO:0005701(IDA)
ShNF-YC15	nucleus	lipid particle GO:0005811(IDA); nucleosome GO:0000786(IDA); nucleus GO:0005634(IDA); polytene chromosome chromocenter GO:0005701(IDA
ShNF-YC16	nucleus	CCAAT-binding factor complex GO:0016602(IEA); nucleoplasm GO:0005654(TAS); transcription factor complex GO:0005667(IC)
ShNF-YC17	nucleus	CCAAT-binding factor complex GO:0016602(IEA); nucleoplasm GO:0005654(TAS); transcription factor complex GO:0005667(IC
ShNF-YC18	nucleus	CCAAT-binding factor complex GO:0016602(IEA)
ShNF-YC19	nucleus	CCAAT-binding factor complex GO:0016602(IEA); nucleoplasm GO:0005654(TAS); transcription factor complex GO:0005667(IC);
ShNF-YC20	nucleus	CCAAT-binding factor complex GO:0016602(IDA); nucleoplasm GO:0005654(TAS)
ShNF-YC21	nucleus	CCAAT-binding factor complex GO:0016602(IDA); nucleoplasm GO:0005654(TAS)
ShNF-YC22	nucleus	CCAAT-binding factor complex GO:0016602(IDA)
ShNF-YC23	nucleus	CCAAT-binding factor complex GO:0016602(IEA); chromatin GO:0000785(NAS); cytoplasm GO:0005737(IDA); nucleus GO:0005634(IDA)
ShNF-YC24	nucleus	nucleosome GO:0000786(IEA); nucleus GO:0005634(IEA)
